# News and Miscellany

**Published:** 1901-04

**Authors:** 


### News and Miscellany.

---

**Dont** fail to go to Galveston, April 23rd. See program 33rd Annual Meeting, State Medical Association.

---

**Married** at Purcell, I. T., March 18th (ult.), Dr. J. J. Eargle, of Proctor, Texas, to Miss Louis Lee, of Lexington, Okla.

---

**Married** at Austin, Texas, April 2nd, inst., Dr. O. H. Radkey, of Manor, Texas (U. T., 1900), to Miss Sadie Hewlett, of Austin, Texas.

---

**The New Medical Practice Act of Texas** will be printed in pampblet form and furnished, postpaid, for 20 cents each.—Daniel.

---

**All My Able Editorials** are knocked out this month, to make room for more interesting matter. Will make it good when fly-time comes.

---

**Dr. C. B. Williams,** of Mineral Wells, is taking a special course in eye, ear, nose and throat at the New Orleans Polyclinic. Dr. G. W. Akard, of Springtown, Texas, is also at the Polyclinic.

---

**Dr. J. M. Fry,** of Wills Point, suffered the loss of his residence by the cyclone which struck that city March 9th (ult.). Mrs.

Fry, Sr., and Dr. H. T. Fry were painfully injured. Dr. Fry, Sr., was painfully but not seriously hurt. Other members of the family escaped unhurt.

---

**Must Vaccinate.**—In a test case made here in Austin, the Attorney General has decided that school trustees have the right to require certificate of successful vaccination of all children making application to be admitted to schools. Now let every county in Texas require it, and the battle will be half won.

---

**I want** a few copies of the January and March numbers of the JOURNAL and will pay 10 cents each for them, or advance subscription correspondingly if sent by subscribers. The demand exceeded the supply and cleaned me out. Had, actually, to refund money (not much)—contrary to all rule and precedent—because I couldn't fill orders for March number.

---

**Ho, for Galveston!**—Reduced rates from I. & G. N. points to Galveston and return will be in effect at all stations, account meeting of the Texas State Medical Association, to convene at Galveston, April 23–26. The well known superior passenger service of the I. & G. N. may be relied upon by those contemplating attending this meeting. Any I. & G. N. agent will be glad to furnish particular information in regard to this matter.

---

**Attention!**—For the Confederate Veterans' Reunion at Memphis, Tenn., May 28th to 30th, inclusive, the Texas & Pacific Railway Company will sell round trip tickets at very low rates. On sale at stations west of Big Springs, May 24th, 25th and 26th. At stations Big Springs and east, in Texas, May 25th, 26th and 27th, final limit, to leave Memphis as late as June 4th, 1901. Extension to June 19th, 1901, on payment of 50 cents and deposit of ticket on or before June 2nd with joint agent at Memphis.

For particulars, regarding rates, sleeping cars, chair cars, etc., see nearest ticket agent, or write Mr. H. P. Hughes, Traveling Passenger Agent, Fort Worth, or Mr. E. P. Turner, G. P. & T. A., Dallas, Texas.

Will be glad to hear from you.

